# News and Miscellany

**Published:** 1900-12

**Authors:** 


### News and Miscellany.

**Died.**—At Ammannsville, Texas, November 13, 1900, Ludmila, daughter of Dr. J. S. Zvesper and wife, Anna, aged 14 months.

**Eor Sale.**—Good practice in small railroad town in good, black land farming country. Collections good. Have three churches; good school. Cost of property will secure the practice. Address, Box 44, Pendletonville, Texas.

**New Orleans Polyclinic.**—Physicians will find the Polyclinic an excellent means for posting themselves upon modern progress in all branches of medicine and surgery. The specialties are fully taught, particularly laboratory work. Fourteenth annual session opens November 12, 1900. For further information address Dr. Isadore Dyer, Secretary, New Orleans Polyclinic, New Orleans.

---

**Early** to bed and early to rise does very well for preachers and guys, but makes a man miss all the fun when he dies and join the old stiffs that are up in the skies. Go to bed when you please, and lie at your ease, and you'll die just the same from a Latin disease. —*Monthly Retrospect.*

---

**Mr. Chas. L. Trusler,** general manager for the McCoy-Howe Co., chemists of Indianapolis, favored us with a pleasant visit last week and showed us the beauties and advantages of *Antiseptic Sphenoids*, the firm's great specialty. Look out for their advertisement next issue: "Like an entering wedge."

---

**Flannel underwear** should be relegated to the attic of antiquity, along with the feather bed and upholstered furniture. It is irrational; it keeps one with a bad cold all the time. The Deimel Linen Mesh Underwear, heavy enough for winter, fills the requirements for comfort and hygiene. See advertisement.

---

**At the last** meeting of the St. Louis Academy of Medical and Surgical Sciences, the following officers were elected for 1901: President, Dr. Emory Lamphear, senior vice-president, Dr. Carl Pesold; junior vice-president, Dr. H. S. P. Lare; secretary, Dr. O. L. Suggett; treasurer, Dr. G. M. Phillips; Orator, Dr. Wm. Porter; librarian, Dr. H. G. Nicks.

---

**For the Pan-American Medical Congress.**—The Southern Pacific Company (Morgan Line) offer a round trip rate of $50, including berth and meals on the steamer. The latter leaves New Orleans December 17 and 22, returning as early as December 31, to arrive at New Orleans January 3. We hope a good number will avail themselves of this opportunity.

**For Headache.**—Sharpe & Dohme make a preparation which we have found safe and efficient for headache. It is called *Anodyne Compound* and consists of Acetanilid, Caffeine and Soda bi-Carb, in 5 grain tablets. Try it, ye weary workers.

---

**Prize Contest.**—The *Southern Medical Journal* is offering a Zentmayer, $100.00 microscope, with all accessories for making any examination required in the practice of medicine, and a Universal Crandall, $75.00 typewriter, for the best two original essays on any subject pertaining to medicine or surgery. Contest closes May 1, 1901. Write for particulars.—*Southern Medical Journal*, LaGrange, N. C.

---

**Mosquito Causes Yellow Fever.**—It is understood that a report concerning the investigations of the acute infectious diseases prevalent in Cuba will soon be made to Surgeon General Sternberg. This report, it is declared, will show that mosquitos are largely responsible for the spread of yellow fever, the germs of which had been injected into his system by a mosquito that had bitten a person afflicted with yellow fever. It will also show, it is said, that another physician who experimented in a similar manner was stricken with yellow fever, but recovered. Dr. Jesse W. Lazear is said to have been the physician who succumbed to the disease, and Dr. James Carroll was the one who recovered. Drs. Carroll and Lazear were stationed in Cuba at the time of the experiments.

---

**Change of Address.**—Dr. T. J. Dodson has removed from Sonora, Texas, to Mangum, O. T. Dr. R. H. Knolle has removed from Schulenburg to New Ulm. Dr. L. M. Berg, late of Laredo, Texas, after having spent a year at the Polyclinics in New York and Chicago, has returned to Texas and located at San Antonio. Dr. W. T. Jones has removed from Marfa to Fort Davis. Dr. W. A. Lockett has removed from Brenham to Amarillo, Texas. Dr. H. H. Brown has removed from Yorktown to Yoakum, Texas, and succeeds Dr. W. T. Jones, late of that place, now of Fort Davis, Texas, as surgeon on the Yoakum division of the S. A. & A. P. R. R. Dr. E. E. Scott has removed from Hanley to Hardeman, Texas. Dr. E. L. Wedemeyer, formerly of Buckhorn, Texas, has purchased the résidence of Dr. H. Upshaw at Gay Hill, where he will practice his profession together with his brother, Dr. G. A. Wedemeyer, under the firm of Drs. Wedemeyer Bros.

**That Bad Mosquito Again.**—Dr. Menger sends us the following additional letter: "According to a late issue of a medical periodical, the result of the Roman Campagna mosquito-experiments is published in the *British Medical Journal.* The result shows that the mosquitos carry malaria, and the following are quotations from the *Journal:* "Drs. Low, Sambon and Sig. Terri and their servants have succeeded in proving that the malaria of the worst part of the Roman Campagna is conveyed by mosquitos. They lived in the worst section of the swamp from June till October which is the most actively malarial period of the year, without becoming at all infected, though it is usually almost fatal to remain there over night. They lived in a mosquito-proof hut. Similar experiments with like results have taken place under Dr. Elliott, a member of the Liverpool expedition to Nigeria, Africa. On the other hand, Dr. Sambon sent carefully selected mosquitos from the Campagna to the London school of tropical medicine, where they were applied to the son of Dr. Manson, who had not been exposed to malarial infection since childhood, with ensuing double tertian intermittent fever." The next thing in order is to banish the malarial mosquito. One great source of the mosquito pest are stagnating water places, especially where weeds abound, and the more weeds in a city the more mosquitoes will be found; it therefore should be a warning to keep the premises and surroundings clear of weeds and water pools; they are the breeding places of mosquitos and malaria.

